# Structural basis for GLP-1 receptor activation by LY3502970, an orally active nonpeptide agonist

**DOI:** 10.1073/pnas.2014879117

**Published:** 2020-11-11

**Authors:** Takahiro Kawai, Bingfa Sun, Hitoshi Yoshino, Dan Feng, Yoshiyuki Suzuki, Masanori Fukazawa, Shunsuke Nagao, David B. Wainscott, Aaron D. Showalter, Brian A. Droz, Tong Sun Kobilka, Matthew P. Coghlan, Francis S. Willard, Yoshiki Kawabe, Brian K. Kobilka, Kyle W. Sloop

**Affiliations:** ^a^Research Division, Chugai Pharmaceutical Co., Ltd., Gotemba, Shizuoka 412-8513, Japan;; ^b^ConfometRx, Santa Clara, CA 95054;; ^c^Quantitative Biology, Lilly Research Laboratories, Eli Lilly and Company, Indianapolis, IN 46285;; ^d^Diabetes and Complications, Lilly Research Laboratories, Eli Lilly and Company, Indianapolis, IN 46285

**Keywords:** LY3502970, OWL833, cryoelectron microscopy, glucagon-like peptide-1 receptor, type 2 diabetes mellitus

## Abstract

Glucagon-like peptide-1 receptor (GLP-1R) agonists are efficacious antidiabetic medications that work by enhancing glucose-dependent insulin secretion and improving energy balance. Currently approved GLP-1R agonists are peptide based, and it has proven difficult to obtain small-molecule activators possessing optimal pharmaceutical properties. We report the discovery and mechanism of action of LY3502970 (OWL833), a nonpeptide GLP-1R agonist. LY3502970 is a partial agonist, biased toward G protein activation over β-arrestin recruitment at the GLP-1R. The molecule is highly potent and selective against other class B G protein–coupled receptors (GPCRs) with a pharmacokinetic profile favorable for oral administration. A high-resolution structure of LY3502970 in complex with active-state GLP-1R revealed a unique binding pocket in the upper helical bundle where the compound is bound by the extracellular domain (ECD), extracellular loop 2, and transmembrane helices 1, 2, 3, and 7. This mechanism creates a distinct receptor conformation that may explain the partial agonism and biased signaling of the compound. Further, interaction between LY3502970 and the primate-specific Trp33 of the ECD informs species selective activity for the molecule. In efficacy studies, oral administration of LY3502970 resulted in glucose lowering in humanized GLP-1R transgenic mice and insulinotropic and hypophagic effects in nonhuman primates, demonstrating an effect size in both models comparable to injectable exenatide. Together, this work determined the molecular basis for the activity of an oral agent being developed for the treatment of type 2 diabetes mellitus, offering insights into the activation of class B GPCRs by nonpeptide ligands.

## Significance

Glucagon-like peptide-1 receptor agonists have become established as a leading class of diabetes medications. However, these peptide-based drugs are administered by subcutaneous injection or, in one case, by a complex oral dosing regimen. We now report the discovery of LY3502970, a potent and selective small-molecule GLP-1R agonist. LY3502970 exhibits preclinical pharmacology equivalent to a marketed injectable GLP-1R agonist and possesses pharmacokinetic properties compatible with oral dosing in humans. Cryoelectron microscopy (cryo-EM) studies reveal an ECD-driven receptor binding mode for LY3502970 that provides a favorable pharmacological profile.

The glucagon-like peptide-1 receptor (GLP-1R) is a member of the class B family of peptide hormone G protein–coupled receptors (GPCRs). The hallmark structural and functional feature of these receptors is a large N-terminal extracellular domain (ECD) (1). The ECD is a globular structure forming trilayer α-β-βα folds that are stabilized by three conserved pairs of cysteine disulfide bonds. These domains play a critical role in class B receptor activation by recognizing the ligand in the initial binding event (1). For the GLP-1R, high-resolution cryoelectron microscopy (cryo-EM) studies show the ECD structure is in an extended open conformation where multiple interactions with the C terminus of GLP-1 occur along a peptide-binding groove (2). These interactions allow the N terminus of GLP-1 to access a deep pocket, thereby rearranging the helical bundle and affecting transmembrane movement to enable interaction with the G protein. This activation mechanism is remarkably efficient as very low concentrations of ligand are needed to elicit an endogenous or a therapeutic response.

Physiologically, upon nutrient ingestion by feeding, GLP-1 is released from intestinal l-cells into the circulation at concentrations in the low picomolar range (5 to 15 pmol/L) (3). These levels of GLP-1 help activate pancreatic β-cell GLP-1R to enhance glucose-stimulated insulin secretion, part of the incretin effect. Therapeutically, the GLP-1 mimetic exenatide was the first GLP-1R agonist approved for the treatment of type 2 diabetes mellitus (T2DM) (4, 5). Exenatide is equipotent with native GLP-1 for activating the GLP-1R (6) and displays beneficial effects on glucose control and body weight reduction at plasma concentrations in the 40 to 70 pmol/L range (7, 8). Similarly, other highly potent GLP-1R agonists, including liraglutide (9), dulaglutide (10), and semaglutide (11), are approved T2DM medications, each with proven cardiovascular health benefits (12). While these medicines provide improved treatment outcomes for patients, all of them are large–molecular weight peptide-based agents that require administration by subcutaneous (s.c.) injection.

An important advance in GLP-1 therapy is the recent development of a coformulated tablet of semaglutide with the absorption enhancer sodium *N*-(8-[2-hydroxybenzoyl] amino) caprylate for oral delivery (13). Although oral bioavailability is less than 1% (14), this approach is therapeutically feasible because of the strong potency of peptide agonists for GLP-1R activation. However, the approved doses of oral semaglutide (brand name Rybelsus) do not achieve the higher drug exposures required to deliver equivalent glucose and body weight–lowering efficacy observed with injectable semaglutide (brand name Ozempic) (14–16). Further, the dosing regimen for oral semaglutide is restrictive for patients since drug absorption is significantly affected by food and fluid in the stomach (17). Specifically, the tablet must be administered after overnight fasting with a proscribed volume of water and at least 30 min before consumption of breakfast or other medicines (14). As an alternative approach, nonpeptide agonists could offer more standard drug formulations with simpler dosing practices, which would be especially beneficial for T2DM patients who often require additional daily medications.

Historically, several groups have attempted to discover small-molecule activators of the GLP-1R, including positive allosteric modulators and compounds with agonist properties. Various chemotypes have been reported, such as series of quinoxalines (18, 19), sulfonylthiophenes (20), pyrimidines (21), phenylalanine derivatives (22), Boc-5 (23), and azoanthracene and oxadiazoanthracene derivatives (24–27). Although this collection of molecules indicated that nonpeptide ligands can modulate GLP-1R activity, these compounds evidently lack potency and pharmacokinetic properties that would be necessary to achieve an efficacy profile similar to that of peptide-based GLP-1R drugs. Therefore, it was hypothesized that small molecules cannot make sufficient contacts throughout the peptide-binding pocket to potently activate the GLP-1R. Here, we challenge this notion by reporting the discovery of a unique ECD-driven binding mechanism for LY3502970, an orally bioavailable nonpeptide agonist of the GLP-1R. This mechanism provides a potent pharmacological profile in vitro and in vivo, supporting the promise of an orally administered GLP-1R agonist drug.

## Results and Discussion

### In Vitro and In Vivo Activity of LY3502970.

**Fig. 1.**
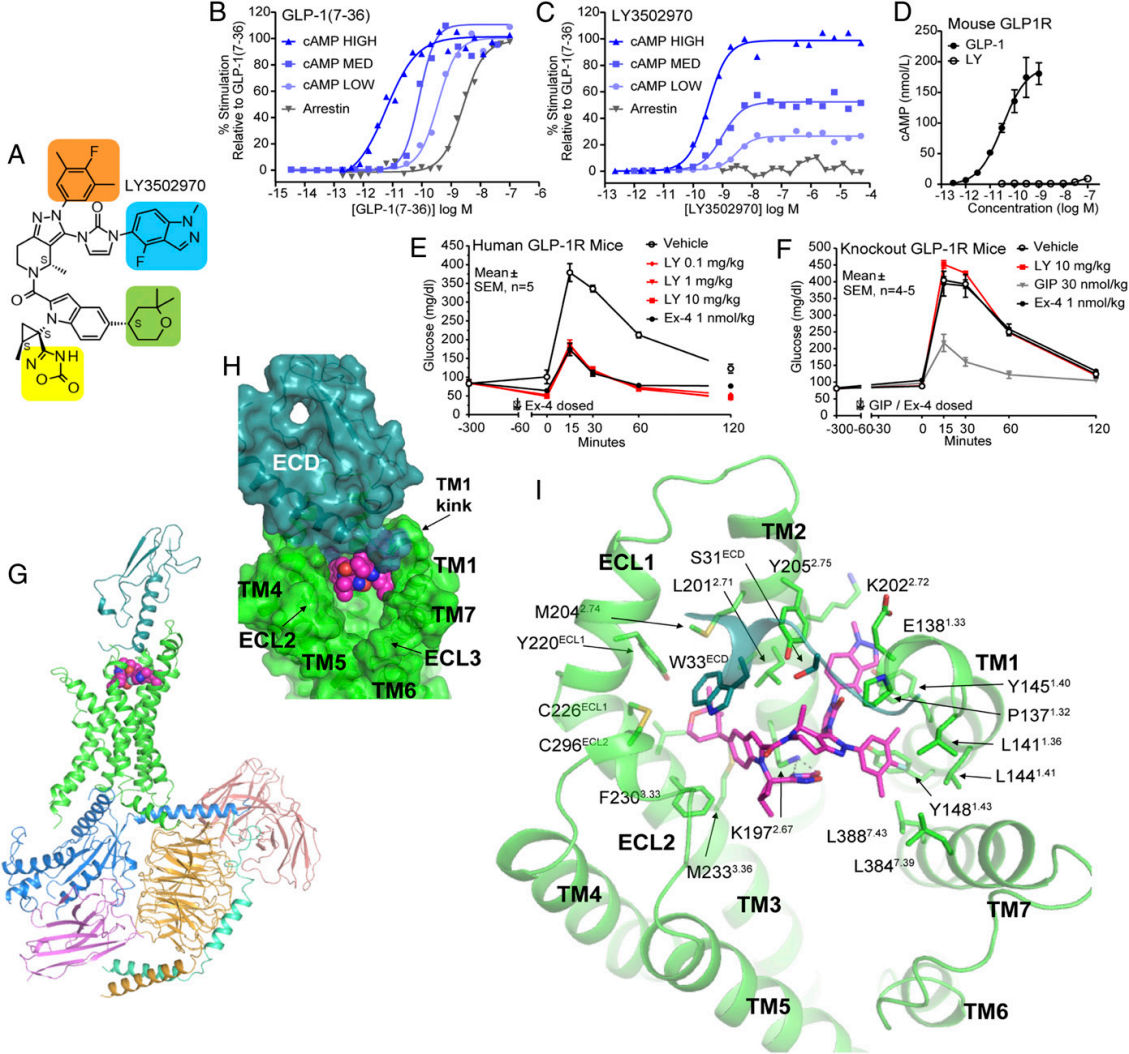
Pharmacology and structural analysis of LY3502970 in complex with GLP-1R/G_siN18_/Nb35/scFv16. (*A*) Chemical structure of LY3502970 (molecular weight: 882.96, formula: C_48_H_48_F_2_N_10_O_5_). Moieties that are discussed in the cryo-EM structural analysis are highlighted in colored boxes. (*B* and *C*) The signal transduction pharmacology of LY3502970 and GLP-1(7-36) was determined. Human GLP-1R density-dependent pharmacology of ligands was quantified by measuring the potency and efficacy for cAMP accumulation at increasing levels of receptor density (high, medium, low). Functional potency and efficacy for β-arrestin recruitment using enzyme fragment complementation was determined. Representative concentration response curves are presented. Summarized data with statistics are presented in *SI Appendix*, Table S1. (*D*) LY3502970 does not stimulate cAMP accumulation in HEK293 cells expressing the mouse GLP-1R. Data are presented as the mean ± SD of three independent experiments. (*E*) Mice expressing the human GLP-1R (*n* = 5 mice/group) were fasted overnight and orally administered vehicle or LY3502970 (0.1 to 10 mg/kg). Five hours later, animals received an intraperitoneal (i.p.) injection of glucose (2 g/kg). As a control group, one cohort was dosed with a s.c. injection of exenatide (1 nmol/kg) 1 h prior to receiving the i.p. glucose. For all mice, the circulating concentration of glucose over various time points was measured using glucometers. Each dose of LY3502970 reduced the glucose excursion AUC versus vehicle (*P* < 0.05; one-way ANOVA followed by the Dunnett’s test). (*F*) Similar studies were perfumed in *Glp1r* null mice. In these experiments, vehicle or LY3502970 (10 mg/kg) was administered orally and exenatide (1 nmol/kg) or gastric inhibitory polypeptide, (GIP) (30 nmol/kg) was dosed via s.c. injection. Data are mean ± SEM (*n* = 4 to 5 mice/group). (*G*) Overall structure of GLP-1R/LY3502970/G_siN18_/Nb35/scFv16. Each subunit or ligand is shown with a different color (GLP-1R: green for 7TM and blue green for ECD; LY3502970: magenta; G_sαiN18_: bright blue; Gβ: light orange; Gγ: green cyan; scFv16: salmon; Nb35: violet). (*H*) Surface representation of LY3502970 binding pocket. The 7TM domain of GLP-1R is colored in green, while ECD is colored blue green. LY3502970 is shown by spheres. (*I*) LY3502970 interacts with residues from both the ECD and 7TM of GLP-1R. LY3502970, and its interaction residues are shown by sticks. Hydrogen bonds are indicated by dashed lines.

Small-molecule activators of the GLP-1R were identified using a screening method that detects compound-induced expression of a urokinase-type plasminogen activator in LLC-PK1 cells (28) expressing the human GLP-1R. Multiple cycles of traditional structure activity relationship work were conducted to optimize affinity and drug-like properties that enabled the discovery of OWL833 (LY3502970) (Fig. 1*A*) (29). Pharmacological studies using HEK293 cells expressing various densities of the human GLP-1R revealed that LY3502970 is highly potent at stimulating GLP-1R-induced cAMP accumulation with partial agonist activity relative to native GLP-1 (Fig. 1 *B* and *C* and *SI Appendix*, Table S1). Further, no detectable recruitment of GLP-1R-mediated β-arrestin was observed for LY3502970 (Fig. 1 *B* and *C*), a feature that may enhance GLP-1R-induced glucose lowering and body weight reduction (30, 31). Although the biased pharmacology is reminiscent of that observed for the nonpeptide ligand TT-OAD2 (32), the potency and efficacy of LY3502970 to stimulate GLP-1R-mediated cAMP accumulation in cell lines expressing different densities of the receptor are far greater than TT-OAD2 (*SI Appendix*, Fig. S1*A* and Table S1). LY3502970 showed no activity on other class B GPCRs (*SI Appendix*, Fig. S1*B*), and strikingly, the compound was also inactive on the mouse (Fig. 1*D*) and other species of the GLP-1R (*SI Appendix*, Fig. S1*C*). Therefore, experiments in mice expressing the human GLP-1R were employed to investigate the in vivo efficacy of LY3502970 (33). In these studies, overnight-fasted animals were orally administered various doses of LY3502970 (0.1 to 10 mg/kg body weight), and 5 h later, the mice were challenged with intraperitoneally injected glucose. Exenatide, the active pharmaceutical ingredient in the registered GLP-1R peptide agonist drugs Byetta and Bydureon, was used as a positive control, but its short half-life required administration (via s.c. injection) 1 h prior to receiving the glucose bolus. LY3502970 demonstrated robust glucose lowering at all doses tested, revealing a potent pharmacodynamic:pharmacokinetic relationship as maximum efficacy was observed with low blood concentrations of the compound (Fig. 1*E*; 10 mg/kg = [1,257 ± 387 nmol/L]_LY3502970_, 1 mg/kg = [205 ± 18 nmol/L]_LY3502970_, 0.1 mg/kg = [24 ± 8 nmol/L]_LY3502970_; mean ± SEM, *n* = 5). These head-to-head experiments with exenatide demonstrate that an orally administered nonpeptide agonist of the GLP-1R can reduce hyperglycemia to the same extent as a registered peptide-based GLP-1 drug. Consistent with the in vitro selectivity data, LY3502970 did not lower glucose in *Glp1r* null mice (Fig. 1*F*). LY3502970 is a low-efficacy, partial agonist for Gα_s_-cAMP activation, yet it exhibits in vivo glucose lowering efficacy comparable to the full agonist exenatide. We propose for the GLP-1 system that a substantial receptor reserve is present in vivo, in that a full biological response can be achieved with partial receptor occupancy (34). Therefore, a partial agonist can induce cAMP pathway activation equivalent to a full agonist in vivo by achieving a higher relative receptor occupancy. For LY3502970, this level of cAMP modulation is achieved in the absence of β-arrestin recruitment, and this may be therapeutically advantageous given emerging hypotheses that G protein–biased GLP-1R agonists have superior efficacy in preclinical models of diabetes (30, 31, 35).

### Structural Basis of GLP-1R Activation by LY3502970.

To investigate the receptor-binding mechanism of LY3502970, cryo-EM was used to determine the structure of the GLP-1R bound to the compound in complex with heterotrimeric G_siN18_, camelid antibody Nb35, and single-chain variable fragment scFv16 at a global nominal resolution of 3.1 to 3.2 Å (Fig. 1*G* and *SI Appendix*, Fig. S2 and Table S2), with well-defined density for each transmembrane (TM) helix, LY3502970, and the majority of the ECD (*SI Appendix*, Figs. S2 and S3). Local resolution near the binding site for LY3502970 is comparable to the overall resolution (2.8 to 3.4Å; *SI Appendix*, Fig. S2*E*), and the well-defined density for the compound allowed confident modeling of the ligand and residues of the pocket (*SI Appendix*, Fig. S3). A positive allosteric modulator was included in the sample preparation to further stabilize the complex; its density was observed near the kinked region of TM6, but the disposition of the potentiator could not be unequivocally determined and was therefore not included in the final model (*SI Appendix*, Fig. S2*F*). We hypothesize that the modulator stabilizes the conformation of TM6, thereby enabling a high-affinity complex for structural studies, but the compound was not included in the in vivo studies that show full glucose lowering by treatment with LY3502970. In the cryo-EM structure, the receptor is in an active conformation, exhibiting typical active-state class B GPCR structure features on the intracellular side, similar to other GLP-1R/G protein complex structures (2, 32, 36) (*SI Appendix*, Fig. S4 *A* and *B*). However, the binding mode of LY3502970 is unique, resulting in a distinct conformation of the ECD and the extracellular portion of the 7TM segments. The compound binds high in the helical bundle, interacting with residues within the ECD, TM1, TM2, TM3, ECL2, and TM7 (Fig. 1 *H* and *I*) but having no interactions with TM4, TM5, and TM6. This contrasts with GLP-1, which interacts with all TM segments except TM4. The binding site of LY3502970 partially overlaps with the area where TT-OAD2 binds (32), although the overall binding modes of these ligands are different (*SI Appendix*, Fig. S5). TT-OAD2 adopts a U-shaped orientation with both ends of its backbone sitting between TM2, TM3, and ECL1 (32). While the 2,2-dimethyl-tetrahydropyran moiety (Fig. 1*A*, green shading) of LY3502970 and the 2,3-dimethylpyridine ring of TT-OAD2 occupy similar positions, the 4-fluoro-1-methyl-indazole moiety (Fig. 1*A*, blue shading) at the other end of LY3502970 extends into the space between TM1 and TM2. Further, the 3,5-dimethyl-4-fluoro-phenyl ring (Fig. 1*A*, orange shading) interacts with TM1 and TM7 (Fig. 1*I* and *SI Appendix*, Fig. S5). In addition to the extensive hydrophobic and aromatic interactions, 4H-1,2,4-oxadiazol-5-one (Fig. 1*A*, yellow shading) of LY3502970 forms strong hydrogen bonds with Lys197^2.67^ (Fig. 1*I*). Together, the conformation of the three branches of LY3502970 establish a unique binding mode for this molecule.

**Fig. 2.**
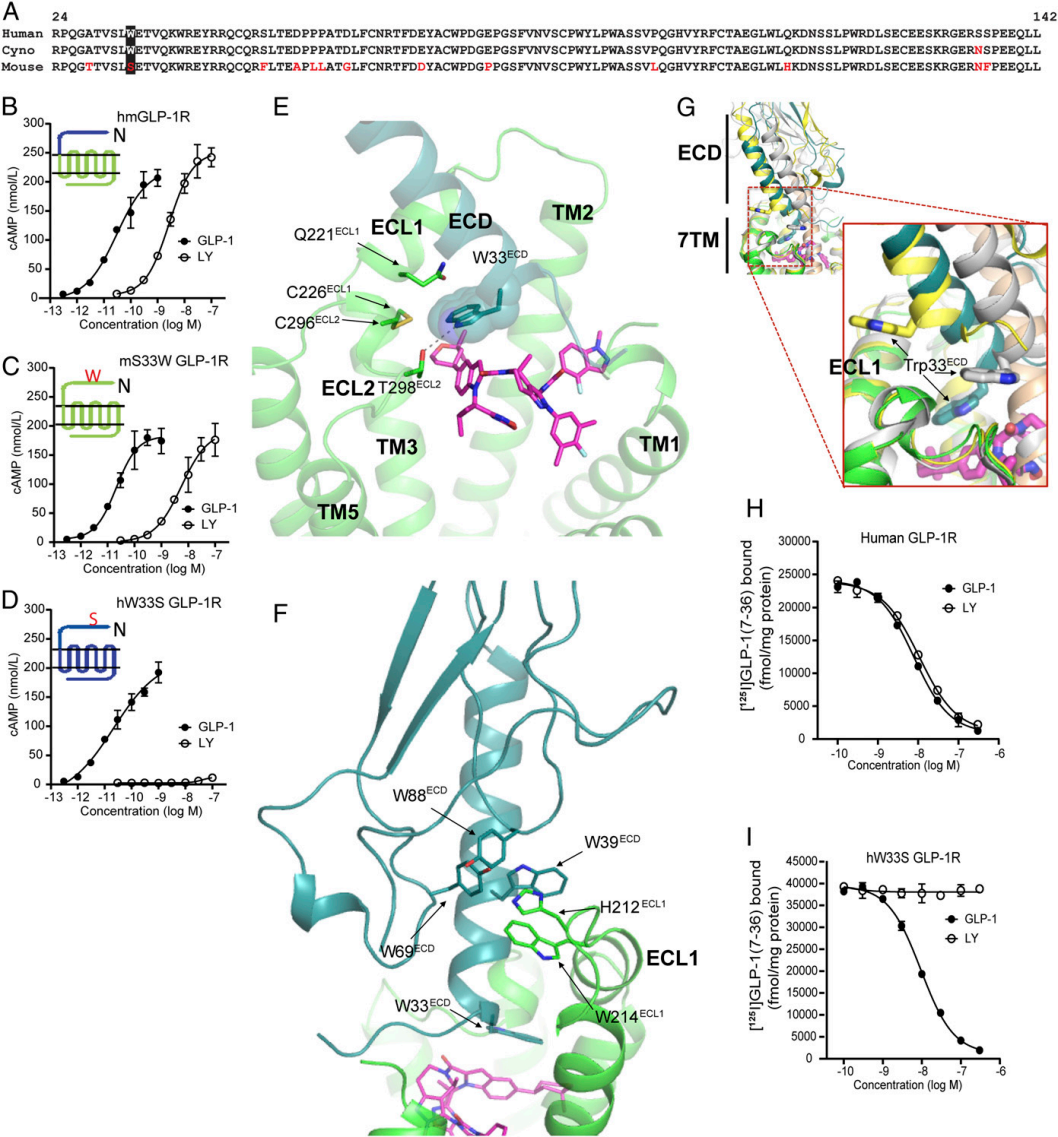
Trp33^ECD^ mediates key interaction with LY3502970 and the surrounding region. (*A*) Amino acid sequence comparison of the ECD regions for GLP-1Rs of different species. (*B*) LY3502970 increases cAMP accumulation in HEK293 cells expressing a chimeric form of the mouse GLP-1R (domains shown in green) where the N-terminal 142 residues have been replaced by the corresponding region of the human GLP-1R (domains shown in blue). (*C*) Mutation of Ser33^ECD^ to Trp33^ECD^ in the mouse GLP-1R enables LY3502970 to stimulate receptor-induced cAMP accumulation, while the reciprocal mutation (*D*) in the human GLP-1R abolishes compound function. The chimera and mutant data are presented as the mean ± SD of three independent experiments. (*E*) Trp33^ECD^ interacts with LY3502970 and residues on TM2, ECL1, and ECL2. Residues and ligands are shown by sticks except Trp33^ECD^, which is also shown by spheres. Hydrogen bonds are indicated by dashed lines. (*F*) GLP-1R ECD orientation in the LY3502970 bound structure stabilized by aromatic interactions with ECL1. (*G*) The unique ECD orientation of ECD in the LY3502970 bound structure as a result of the unique Trp33^ECD^ position. The structures of GLP-1R bound to LY3502970 (7TM in green, ECD in blue green, LY3502970 in magenta), GLP-1 (yellow, PDB ID code 6VCB), and peptide 5 (gray, PDB ID code 5NX2) are aligned. Trp33^ECD^ is shown by a stick. Other peptide bound GLP-1R structures (GLP-1, PDB ID code 5VAI; ExP5, PDB ID code 6B3J) are not shown here, but their ECD orientations are very similar to GLP-1 bound structure 6VCB in yellow. (*H* and *I*) Competitive inhibition of [^125^I]GLP-1(7-36) binding to membranes isolated from cells expressing the human GLP-1R (*H*) or human W33S GLP-1R (*I*). Data are represented as the mean ± SD of three independent experiments.

Domain-swapping experiments investigating the molecular basis for the specificity of LY3502970 activating the human but not the mouse GLP-1R provided functional insight into the receptor activation mechanism of LY3502970. Exchange of the mouse ECD with the corresponding sequence of the human receptor enabled LY3502970 to activate the mouse GLP-1R (Fig. 2*B*). Comparison of the amino acids of the ECD across species shows tryptophan at position 33 (Trp33^ECD^) in primates, while other species have serine at this location (Fig. 2*A*). Site-directed mutagenesis of this residue revealed gain and loss of function in the ability of LY3502970 to activate the mouse and human GLP-1Rs, demonstrating an essential role of Trp33^ECD^ in the receptor activation mechanism of the compound (Fig. 2 *C* and *D*). Indeed, the cryo-EM structure reveals aromatic and hydrophobic interactions between the indole-tetrahydropyran branch (Fig. 1*A*, green shading and the connecting moiety) of LY3502970 and Trp33^ECD^, which forms a lid over this branch of the compound (Figs. 1*I* and 2*E*). The Trp33^ECD^ lid is further stabilized through hydrogen bonding with Thr298^ECL2^ of ECL2 and van der Waals interactions with Gln221^ECL1^ of ECL1 and the disulfide bonding between Cys226^ECL1^ and Cys296^ECL2^ (Fig. 2*E*). The conformation of Trp33^ECD^ is also likely a driving force behind the unique ECD conformation. Other reports have shown that the ECD exhibits a high degree of flexibility and can adopt multiple open or closed conformations (37). In the structure presented here, the ECD is oriented toward ECL1 (Fig. 2 *F* and *G*). An aromatic patch of the ECD consisting of Trp39^ECD^, Tyr69 ^ECD^, and Tyr88 ^ECD^ is packed against His212^ECL1^ and Trp214 ^ECL1^ of the ECL1 (Fig. 2*F* and *SI Appendix*, Fig. S6). In the native GLP-1 and exendin-P5 (ExP5) bound GLP-1R structures, these aromatic patches on the ECD and ECL1 are physically separated by the peptide, engaging in aromatic and hydrophobic interactions with Phe28^GLP1^ (or Phe23^ExP5^), Ile29^GLP1^ (or Ile24^ExP5^), Trp31^GLP1^ (or Trp26^ExP5^), and Leu32^GLP1^ (or Leu27^ExP5^) spanning across the C-terminal region of the peptide (*SI Appendix*, Fig. S6) (2, 36). Taken together, the functional importance of Trp33^ECD^ revealed by the mutagenesis experiments is fully supported by the cryo-EM data showing the interaction of Trp33^ECD^ with LY3502970 to stabilize the binding pocket and consistent with an inability of LY3502970 to activate receptors containing Ser33^ECD^. Competition binding experiments further substantiated this mechanism as Trp33^ECD^ was determined to be necessary for GLP-1R binding of LY3502970 (Fig. 2 *H* and *I*). More broadly, although Trp33^ECD^ is not critical for receptor activation by native GLP-1, additional experiments revealed that another nonpeptide agonist PF-06882961 (38, 39) also requires Trp33^ECD^ for GLP-1R activity (*SI Appendix*, Fig. S7 *A* and *B*).

**Fig. 3.**
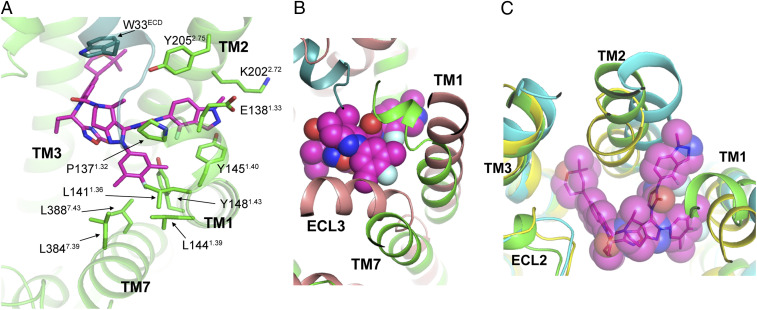
LY3502970 binding and the conformation of TM1, TM2, and TM7 of the GLP-1R. (*A*) LY3502970 (magenta) interactions with TM1, TM2, and TM7 of GLP-1R (green). Residues and ligands are shown by sticks. (*B*) TM1, TM7, and ECL3 of the inactive state structure of GLP-1R (salmon, PDB ID code 6LN2) need to shift to interact with LY3502970 (magenta sphere). (*C*) Unique conformation of TM1 and TM2 to accommodate LY3502970. The structures of GLP-1R bound to LY3502970, GLP-1 (yellow, PDB ID code 6VCB), and TT-OAD2 (cyan, PDB ID code 6ORV) are aligned. Other peptide bound GLP-1R structures (GLP-1, PDB ID code 5VAI; ExP5, PDB ID code 6B3J; peptide 5, PDB ID code 5NX2) are not shown here, but their TM1, TM2, and TM3 conformations are very similar to GLP-1 bound structure 6VCB in yellow.

On the other side of the binding pocket, the 4-fluoro-1-methyl-indazole moiety (Fig. 1*A*, blue shading) of LY3502970 has aromatic interactions with Tyr205^2.75^ and Tyr145^1.40^ (Fig. 3*A*). The interaction with Tyr145^1.40^ is reminiscent of the interaction mode of a GLP-1R-positive allosteric modulator, LSN3160440 (40) (*SI Appendix*, Fig. S8). In close vicinity, the 3,5-dimethyl-4-fluoro-phenyl ring (Fig. 1*A*, orange shading) of LY3502970 interacts with residues on TM1 (Leu141^1.36^, Leu144^1.39^, Tyr148^1.43^) and TM7 (Leu384^7.39^, Leu388^7.43^) (Fig. 3*A*). To accommodate the interaction and avoid steric hindrance, TM7 moves outward, and TM1 moves toward the displaced TM7, in comparison with their positions in the inactive-state GLP-1R structure (37) (Fig. 3*B*). These features are common among all active-state GLP-1R structures (2, 32, 36, 41) and may provide further insight into the mechanism of GLP-1R activation by LY3502970. Another interesting feature is that the extracellular end of TM1 forms a kink from Leu141^1.36^, which is a conformation not observed in any other GLP-1R structure (Fig. 3 *A* and *B* and *SI Appendix*, Fig. S9), suggesting it is the result of the unique binding mode of LY3502970. Together, the conformation of Trp33^ECD^, the preceding N terminus of the ECD, and the kink conformation of the extracellular end of TM1 form a lid to partially bury LY3502970 in the GLP-1R helical bundle (Fig. 1*H*).

In addition to the orientation of the ECD and the kink in TM1, the binding mode of LY3502970 also plays an important role in the distinct conformation of other extracellular regions of the GLP-1R. Alignment of all available GLP-1R structures bound to various ligands shows that the conformations of TM3, TM4, and TM5 are relatively consistent (*SI Appendix*, Fig. S10), while TM1, TM2, TM6, and TM7 vary significantly when bound to different ligands (or without ligand). In the LY3502970 structure, TM2 is positioned farther apart from TM3 than in the native GLP-1 or ExP5 bound structures (2, 36) but closer to TM3 than in the TT-OAD2 bound structure (32) (Fig. 3*C*). Thus, there is enough space for the binding of both the 2,2-dimethyl-tetrahydropyran and the 4-fluoro-1-methyl-indazole moieties of LY3502970 between TM2-TM3 and TM1-TM2, respectively. The unique conformation of TM2 observed in our structure and the binding mode of LY3502970 would be difficult to computationally predict even with prior structural information on GLP-1R, highlighting the conformational flexibility of GLP-1R and GPCRs in general.

**Fig. 4.**
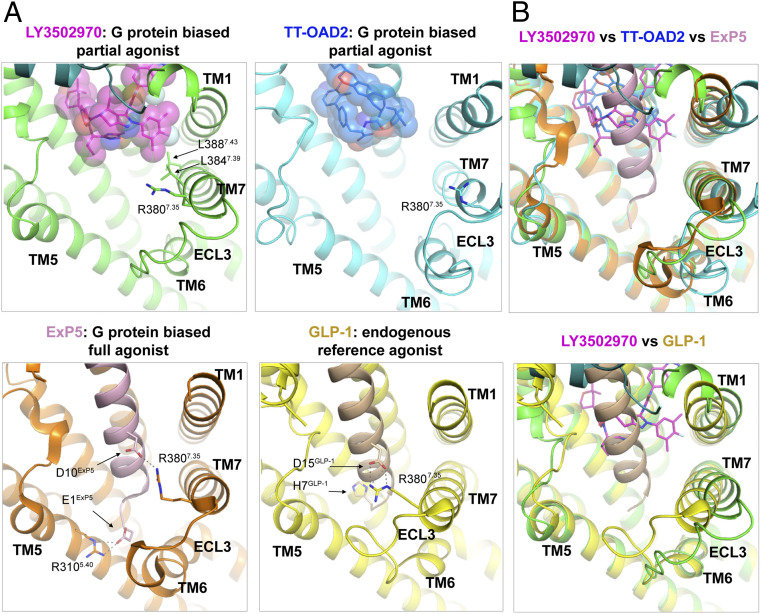
Structures of GLP-1R bound to agonists with different signaling profiles shed light on the structural basis for partial agonism and biased signaling. (*A*) Structures of the GLP-1R bound to LY3502970 (7TM in green, LY3502970 shown by magenta spheres), native GLP-1 (receptor in yellow, GLP-1 in beige, PDB ID code 6VCB), ExP5 (receptor in orange, ExP5 in pink, PDB ID code 6B3J), or TT-OAD2 (receptor in cyan, TT-OAD2 in blue, PDB ID code 6ORV) are aligned and shown from an identical view. Critical residues are shown by sticks. Hydrogen bonds are indicated by dashed lines. (*B*) The structure of GLP-1R bound to LY3502970 is aligned with GLP-1R bound to ExP5 and TT-OAD2 (*Upper*) and GLP-1 (*Lower*).

Several structures of the GLP-1R bound to various agonists that show different signal transduction properties have been reported, and structural analysis sheds light on the potential structural determinants of biased signaling and partial agonism. Compared with native GLP-1, other ligands that exhibit bias toward the G protein–dependent cAMP pathway are associated with a distinct conformation of TM7 at its extracellular end (Fig. 4 *A* and *B*). Upon superimposition, the position of the Cαs of Arg380^7.35^ in the LY3502970, ExP5, and TT-OAD2 bound GLP-1R structures is shifted from its position in the GLP-1 bound structure by 4, 4, and 10 Å, respectively, away from the consistently aligned TM5 (Fig. 4 *A* and *B*). This conformational difference can be directly attributed to the ligands’ mode of interaction with TM7 (Fig. 4*A*). Therefore, close positioning of the extracellular portion of TM7 and TM5 in an active conformation may be required for efficient β-arrestin recruitment. Compared with GLP-1 and ExP5, both of which are full agonists for cAMP signaling, the two partial agonists, LY3502970 and TT-OAD2, both lack interaction with Arg380^7.35^ or any other interaction to stabilize the conformation of the extracellular portion of the TM6-ECL3-TM7 above Arg380^7.35^ (Fig. 4*A*). As mentioned above, LY3502970 interacts with TM7 (Fig. 3*A*), while TT-OAD2 does not have any direct interactions with TM7. This difference may explain the greater potency of LY3502970 over that for TT-OAD2 in adenylyl cyclase activation (Fig. 1*C* and *SI Appendix*, Fig. S1*A* and Table S1). Overall, these analyses suggest that stabilizing the conformation of the TM6-ECL3-TM7 region may be required for full, unbiased agonism.

### Pharmacokinetics and Function in Cynomolgus Monkeys.

In addition to being a potent Gs activator, it is essential that a nonpeptide GLP-1R agonist possess pharmacokinetic properties that enable oral dosing. Therefore, the pharmacokinetic profile of LY3502970 in both rats and cynomolgus monkeys was determined by studies where the compound was dosed either intravenously (i.v.) or orally. The elimination half-life following oral administration (T_1/2_) was 10.4 to 12.4 h in rats (*n* = 4) and 3.4 to 4.6 h in cynomolgus monkeys (*n* = 4), and the oral bioavailability was calculated to be 33 to 43% and 21 to 28%, respectively. This contrasts with the 0.4 to 1% oral bioavailability reported in humans for the only peptide GLP-1R agonist tablet approved to date (14). These data suggest that oral administration of LY3502970 may be feasible in the absence of complex oral formulations that are required for peptide-based GLP-1R agonists (17).

**Fig. 5.**
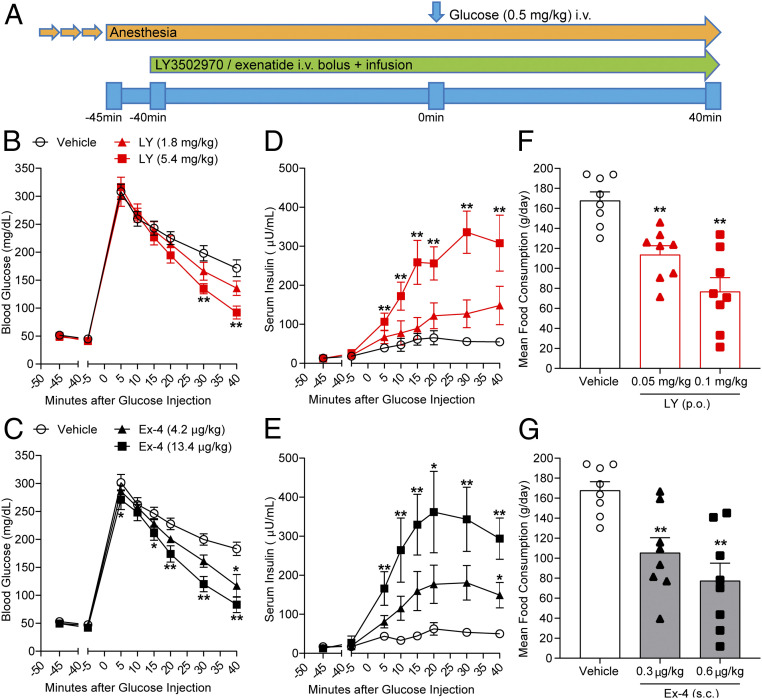
LY3502970 enhances glucose-stimulated insulin secretion and reduces food consumption in cynomolgus monkeys. (*B*–*E*) Effects of LY3502970 in IVGTT experiments. (*A*) Schematic diagram of the IVGTT procedure in anesthetized cynomolgus monkeys. Dosing of LY3502970 or exenatide was performed by manual bolus injection, followed by continuous infusion for 80 min. Forty minutes after initiating dosing of vehicle or the drug, glucose was administered intravenously. (*B* and *C*) Effect of LY3502970 (*B*) or exenatide (*C*) on blood glucose levels. (*D* and *E*) Effect of LY3502970 (*D*) or exenatide (*E*) on serum insulin levels. Data are represented as the mean ± SEM (*n* = 7). Significantly different from vehicle 1 or vehicle 2 by Dunnett’s test at *P < 0.025 and **P < 0.005 (one tailed), respectively. Vehicle 1: PEG400/PG/Gly NaOH buffer (pH 9), vehicle 2: 0.05% Tween80 PBS. (*F* and *G*) Mean food consumption following administration of LY3502970 (*F*) or exenatide (*G*). Cynomolgus monkeys were administered LY3502970, exenatide, or vehicle once daily for 5 d with a 2 d recovery period using an 8 × 5 cross-over design. Food consumption during the 90 min period after feeding was measured. Data are represented as the mean ± SEM (*n* = 8). Significantly different from the vehicle group by Dunnett’s test at **P < 0.005 (one tailed). Vehicle: dimethyl sulfoxide (DMSO)/Cremophor/PEG400/Gly-NaOH buffer (pH 10) [p.o.] + 0.05% Tween80 PBS [s.c.], LY3502970: LY3502970 [p.o.] + 0.05% Tween 80 PBS [s.c.], exenatide: DMSO/Cremophor/PEG400/Gly-NaOH buffer (pH 10) [p.o.] + exenatide [s.c.].

Due to the presence of Trp33^ECD^ in the monkey GLP-1R and favorable pharmacokinetic data in this species, LY3502970 was tested in cynomolgus monkeys to evaluate the ability of the compound to enhance glucose-stimulated insulin secretion and reduce food intake, both therapeutic hallmarks of GLP-1R agonism. Intravenous glucose tolerance tests (IVGTTs) were conducted to assess the ability of LY3502970 to enhance insulin secretion. The compound or exenatide was i.v. administered, followed by continuous infusion to maintain steady-state drug concentrations during the test. Glucose was administered 40 min after the infusion of LY3502970 or exenatide (Fig. 5*A*). Prior to the glucose administration, neither LY3502970 nor exenatide stimulated insulin secretion. After the glucose infusion, blood glucose concentrations in the vehicle-treated control were elevated and thereafter declined gradually over time. Serum insulin levels were slightly increased and remained elevated for 40 min. Treatment with LY3502970 or exenatide significantly increased the insulin concentrations and lowered blood glucose during the experiment (Fig. 5 *B*–*E*). Insulin secretion effected by the high dose of LY3502970 (steady-state concentration: 9.1 ± 0.8 nmol/L; mean ± SEM, *n* = 7) was comparable to that stimulated by high-dose exenatide (43.0 ± 4.1 pmol/L; mean ± SEM, *n* = 7). These results indicate that LY3502970 can reduce hyperglycemia via an insulinotropic mechanism to an extent similar to exenatide.

Since peptide-based GLP-1R agonists exhibit an anorexigenic effect as part of their overall ability to improve metabolic control, LY3502970 was orally administered to monkeys to examine the ability of the compound to reduce feeding. Following LY3502970 or exenatide treatment, food consumption was measured for 90 min. For these studies, LY3502970 was administered orally 180 min before feeding, and exenatide was s.c. injected 30 min prior to food availability, in line with the time of maximum concentration observed (Tmax) in monkey pharmacokinetic studies. Dosing was conducted once daily for 5 d with a 2 d recovery period. LY3502970 at 0.05 and 0.1 mg/kg decreased food consumption from the first to the fifth days of dosing in a dose-dependent manner (Fig. 5*F*), similar to that observed for 0.3 and 0.6 µg/kg of exenatide (Fig. 5*G*). The mean LY3502970 and exenatide concentrations which decreased food consumption were 8.3 ± 0.8 nmol/L and 83.1 ± 4.5 pmol/L, respectively (mean ± SEM, *n* = 8). These results indicate that orally dosed LY3502970 can achieve a reduction of food intake similar to the injectable GLP-1R agonist, exenatide. Taken together, LY3502970 displays a preclinical pharmacodynamic profile similar to marketed peptide-based GLP-1R agonists and possesses pharmacokinetic properties compatible with oral dosing in humans. Consequently, LY3502970 is currently being evaluated in early stage clinical trials for its potential as an antidiabetic agent (identifier, NCT04426474).

## Methods

### In Vitro Pharmacology.

cAMP accumulation, β-arrestin recruitment, and receptor binding assays were performed as previously described (28, 40).

### Mutant Receptor Construction.

A plasmid expression vector containing a chimeric sequence where the ECD of the human GLP-1R replaced the mouse ECD was generated using the PCR products (PrimeSTAR Max, Takara) introduced to the multicloning site of pCMV6 entry using an In-Fusion HD cloning kit (Takara). The PCR primers used to create these constructs are as follows: for hmGLP-1R, 5′-CCG​GAG​GAG​CAG​CTC​CTGTCC​CTG​TAC​ATT​ATC​TAC‐ACA​GTG​GGG-3′ and 3′-AGG​GGC​CTC​CTC​GTC​GAG​GAC-5′ and, for mhGLP-1R, 5′-TTC​CTC​TAC​ATC​ATC​TAC​ACG​GTG​GGC-3′ and 3′-GAA​AGG​ACT​CCT​TGT​CGA​GGA‐CAAG​GAG​ATG​TAG​TAG​ATG-5′.

Human W33S GLP-1R and mouse S33W GLP-1R plasmids were generated by PCR-based mutagenesis; then the PCR products were introduced to pCMV6 entry described above. The PCR primers used to create these constructs are as follows: for hW33S GLP-1R, 5′-TGT​CCC​TCTCAGAG​ACG​GTG‐CAG​AAA​TGG​C-3′ and 3′-CCC​ACG​GTG​ACA​CAG​GGA​GAGTCTC​TGC​CA-5′ and, for mS33W GLP-1R, 5′-TCC​CTCTGGGAG​ACG​GTG​CAG​AAA​TGG​AGA-3′ and 3′-CCA​TGG​TGC​CAC​AGG​GAGACCCTC​TGC​CAC-5′.

DNA sequencing confirmed the chimera and single-point mutant constructs.

### Cryo-EM Method.

#### GLP-1R/LY3502970/G_siN18_ complex formation and purification.

The GLP-1R/LY3502970/G_siN18_ complex (referred to as “the complex” in the following text) was formed on membrane as previously described (2, 40) with slight modification. The main difference was that we used a modified version of the alpha subunit of the stimulatory G protein, namely, G_siN18_, which has the 1 to 18 residues of G_ai_ at its N terminus, replacing the original 1 to 25 residues of G_as_. This modification does not change any regions on G_as_ that are involved in receptor coupling or nucleotide binding but allows the coupling to scFv16 to aid stabilization of the complex and cryo-EM structure determination (42). To prepare the complex sample, Sf9 cell pellets infected with virus containing human GLP-1R (residues 24 to 422) were lysed, and a membrane sample was collected and washed. The complex was formed by incubating the purified molar excess of G_siN18_, Nb35, scFv16, 10 µM LY3502970, and 2 µM LSN3451217 with membranes and then solubilized in buffer composed of 1% *n*-dodecyl β-d-maltoside (DDM), 0.5% 3-[(3-cholamidopropyl)dimethylammonio]-1-propanesulfonate (CHAPS), 0.2% cholesteryl hemisuccinate, 30 mM Hepes (pH 7.8), 150 mM NaCl, 30% glycerol, 25 µM Tris(2-carboxyethyl)phosphine (TCEP), 2.5 mg/mL leupeptin, 0.16 mg/mL benzamidine, 10 µM LY3502970, and 2 µM LSN3451217. The complex was then purified by affinity chromatography using anti-FLAG M1 resin and exchanged for buffer composed of 30 mM Hepes (pH 7.5), 150 mM NaCl, 2.5 mM CaCl_2_, 10 µM LY3502970 and 2 µM LSN3451217, 25 μM TCEP, 0.25% lauryl maltose neopentyl glycol (MNG, NG310 Anatrace), 0.25% GDN101 (Anatrace), 0.048% 1-palmitoyl-2-oleoyl-sn-glycero-3-phospho-1’-rac-glycerol (POPG, Avanti), and 0.03% cholesterol (Sigma-Aldrich). The complex was eluted and further purified by size exclusion chromatography using a Superdex S200 10/300 GL column with a running buffer of 30 mM Hepes (pH 7.5), 150 mM NaCl, 10 µM LY3502970 and 2 µM LSN3451217, 100 μM TCEP, 0.015% MNG, 0.005% GDN101, 0.00192% POPG, and 0.0012% cholesterol. The fractions for the monomeric complex were collected and concentrated individually for electron microscopy experiments.

#### Cryo-EM data acquisition and processing.

This procedure was carried out as previously described (40). A sample of 3.5 μL of purified GLP-1R/LY3502970/G_siN18_ complex at a concentration of ∼10 mg/mL was applied to glow-discharged holey carbon grids (Quantifoil R1.2/1.3, 200 mesh) and subsequently vitrified using a Vitrobot Mark IV (Thermo Fisher Scientific). The specimen was visualized with a Titan Krios electron microscope (Thermo Fisher Scientific) with an energy filter operating at 300 kV accelerating voltage at a nominal magnification of 130,000× using a K2 Summit direct electron detector (Gatan, Inc.) in counting mode, corresponding to a pixel size of 1.04 Å on the specimen level. A total of 5,427 images was collected, and the statistics of data collection are listed in *SI Appendix*, Table S2.

Data processing was performed in Relion3.0 (43). Dose-fractionated image stacks were subjected to beam-induced motion correction using MotionCor2 (44). Contrast transfer function (CTF) parameters for each micrograph were determined by Gctf (45). Manual selection of micrographs based on their quality and CTF estimated resolution left 3,925 images to proceed to subsequent steps. Particle selection with two-dimensional (2D) and 3D classifications were performed on a binned dataset with a pixel size of 2.08 Å. A total of 1,598,390 particles was initially picked using semiautomated particle selection with a template from a previously reported GLP-1R/GLP-1/G_s_/Nb35 complex (2); these particles were subjected to 2D classification and 3D classification. After 3D classification, 583,053 particles were selected, and unbinned versions of these particles were used as the input for 3D autorefinement. Bayesian polishing and CTF refinement (46) were applied to further improve the map, resulting in a 3.1 Å overall resolution map. The flowchart is shown in *SI Appendix*, Fig. S2*A*. Reported resolution is based on the gold-standard Fourier shell correlation using the 0.143 criterion (*SI Appendix*, Fig. S2*B*).

### Model Building and Refinement.

This procedure was carried out as previously described (40). An initial model was formed by rigid-body fitting of the reported GLP-1R/GLP-1/G_s_/Nb35 structure (Research Collaboratory for Structural Bioinformatics Protein Data Bank [PDB] ID code 5VAI). This starting model was then subjected to iterative rounds of manual and Real Space Refinement in Coot (47, 48) and Phenix (49), respectively. The final model was visually inspected for general fit to the map, and geometry was further evaluated using Molprobity (50). For cross validation, Fourier shell correlation curves were calculated between the resulting model and the half map used for refinement, as well as between the resulting model and the other half map for cross validation, and also against the full final map (*SI Appendix*, Fig. S2*C*). The final refinement statistics for the model are summarized in *SI Appendix*, Table S2.

Atomic coordinates and the cryo-EM density map have been deposited in the PDB under PDB ID code 6XOX and the Electron Microscopy Data Bank (EMDB) entry ID EMD-22283.

### Animal Welfare.

Animals were studied and maintained in accordance with the Institutional Animal Care and Use Committee (IACUC) of Eli Lilly and Company and the Guide for the Use and Care of Laboratory Animals by NIH (51). The studies using mice were approved by the IACUC of Eli Lilly and Company, and experiments using rats and cynomolgus monkeys were approved by the IACUC of Chugai Pharmaceutical Co., Ltd. Pharmacokinetic studies were performed in accordance with animal welfare bylaws of Sekisui Medical Co., Ltd., Drug Development Solutions Division, Drug Development Solutions Center, which is accredited by the Association for Assessment and Accreditation of Laboratory Animal Care (AAALAC) International. The IVGTT and food intake studies were performed in accordance with animal welfare bylaws of Shin Nippon Biomedical Laboratories, Ltd., Drug Safety Research Laboratories, which is also accredited by AAALAC International.

### Compound Formulation.

LY3502970 was prepared in 10% polyethylene glycol 400 (PEG400, Wako Pure Chemical Industries)/10% propylene glycol (PG, Wako Pure Chemical Industries)/80% glycine buffer (100 mM glycine, 64 mM NaOH, pH 10) buffer. Exenatide was prepared in phosphate-buffered saline (PBS) containing 0.05 wt/vol% Tween80 buffer. The vehicle solutions without the test articles were used as controls.

### Pharmacokinetics.

LY3502970 was administered orally at doses of 0.05, 0.15, or 0.45 mg/kg or i.v. at 0.15 mg/kg to 8-wk-old male rats (RccHan: WIST, Japan SLC; *n* = 4 rats/group) or oral doses of 0.04, 0.12, or 0.36 mg/kg or i.v. at 0.12 mg/kg to 3-y-old male cynomolgus monkeys (Hamri Co., Ltd.; *n* = 4 monkeys/group). Blood was collected predose and 30 min and 1, 2, 3, 4, 6, 8, 12, 16, and 24 h after administration in orally dosing group. Blood samples were also collected predose and 2, 10, and 30 min and 1, 2, 4, 8, 12, 16, and 24 h after i.v. administration. Compound concentrations were determined by liquid chromatography–tandem mass spectrometry (AB SCIEX Triple Quad 5500, AB SCIEX), which had a lower limit of quantification of 0.1 ng/mL. Pharmacokinetic parameters were calculated by noncompartmental analysis (linear/log trapezoidal rule) in Phoenix WinNonlin (Version 6.4, Certara). Oral bioavailability (BA) was calculated with area under the concentration-time curve from zero to infinity (AUCinf) after oral and i.v. administration by BA (%) = AUCinf, by mouth, orally (p.o.)/AUCinf, i.v. × 100.

### Glucose Tolerance Tests.

Mice fasted overnight were orally dosed with vehicle or LY3502970, followed 5 h later by an intraperitoneal injection of glucose (2 g/kg). Blood glucose concentrations were measured over time up to 120 min after glucose administration using glucometers. Data were used to calculate the area under the curve (AUC) (*n* = 5 mice/group). Male cynomolgus monkeys (3.9 to 7.5 kg, Shin Nippon Biomedical Laboratories, Ltd.) were administered atropine sulfate i.v. (0.5 mg Tanabe, Mitsubishi Tanabe Pharma Corporation, 0.02 mL/kg) and sedated by an intramuscular injection of ketamine hydrochloride (500 mg, Daiichi Sankyo Propharma, 50 mg/mL, 0.2 mL/kg). Animals were then anesthetized by inhalation of isoflurane (Isoflu, Zoetis Japan, 0.5 to 2.0%) using a ventilator. To maintain steady-state drug concentrations of the test article, dosing of LY3502970 or exenatide was performed by manual bolus injection, followed by continuous infusion for 80 min into the cephalic vein of the forearm or the saphenous vein of the leg by a syringe, indwelling needle, extension tube, three-way stopcock, and syringe pump (BS-8000, Braintree Scientific Inc.). Low and high doses were 1,800 and 5,400 ng/kg, respectively, for LY3502970 and 4.2 and 13.4 ng/kg for exenatide. Dosing volumes were 2 mL/kg for the bolus administration, and the infusion rates for low- and high-dose LY3502970 were 1,280 and 3,840 ng⋅kg^−1^⋅h^−1^ and were 6.5 and 21.8 ng⋅kg^−1^⋅h^−1^ for low- and high-dose exenatide. Infusion volume was 2.7 mL/kg at a speed of 2 mL⋅kg^−1^⋅h^−1^. Forty minutes after initiation of dosing, 40% glucose (Otsuka Pharmaceutical Factory) was administered at 1.25 mL⋅kg^−1^⋅min^−1^ via the cephalic or saphenous vein. Blood was collected from the femoral vein 5 min before and after dosing and then at 5, 10, 15, 20, 30, and 40 min following administration of 40% glucose. The studies were conducted at intervals of 7 or 24 d (days 8, 15, 22, 29, 36, and 60) using a 7 × 6 cross-over design.

### Food Consumption Studies.

Eight male cynomolgus monkeys (7.5 to 9.3 kg, Shin Nippon Biomedical Laboratories, Ltd.) were administered LY3502970, exenatide, or vehicle once daily for 5 d with a 2 d recovery period using an 8 × 5 cross-over design. Food consumption during the 90 min period following presentation of food was measured in animals previously administered LY3502970, exenatide, or vehicle as follows: 1) LY3502970 at 0.05 or 0.1 mg/kg by oral administration 180 min before feeding, 2) exenatide at 0.3 or 0.6 µg/kg by s.c. injection 30 min before feeding, or 3) the matched vehicle administered at the appropriate time.

### Statistical Analysis.

Statistical analyses of circulating glucose, insulin, and food consumption were performed by linier mixed model ANOVA with animal as a random effect and dose level as a fixed effect for comparisons by Dunnett’s test with adjustment for multiplicity and by one-sided significance level of 2.5% for the order of increase (insulin) or decrease (glucose, mean food consumption) using SAS System for Windows, Release 9.2 (SAS Institute Inc.). Quantitative results are represented as the mean ± SEM.

The chemical structure of LY3502970 (OWL833) and some of the pharmacology data are shown in WO2018/056453.

## Supplementary Material

Supplementary File

## Data Availability

Atomic coordinates and the cryo-EM density map have been deposited in the Protein Data Bank (PDB ID code 6XOX) and EMDB (entry ID EMD-22283).
