# Supplementary material for: Structural basis for GLP-1 receptor activation by LY3502970, an orally active nonpeptide agonist
Source: Proc Natl Acad Sci U S A. 2020 Nov 11;117(47):29959–67. doi: 10.1073/pnas.2014879117 (PMC7703558; doi:10.1073/pnas.2014879117)
Supplement: Supplementary File [file pnas.2014879117.sapp.pdf]

Title: Structural basis for GLP-1 receptor activation by LY3502970, an orally active non-peptide agonist

Takahiro Kawai[1¶], Bingfa Sun[2¶], Hitoshi Yoshino[1¶], Dan Feng[2], Yoshiyuki Suzuki[1*], Masanori Fukazawa[1], Shunsuke Nagao[1], David B. Wainscott[3], Aaron D. Showalter[4], Brian A. Droz[4], Tong Sun Kobilka[2], Matthew P. Coghlan[4], Francis S. Willard[3], Yoshiki Kawabe[1], Brian K. Kobilka[2#*], and Kyle W. Sloop[4*]

[1]Research Division, Chugai Pharmaceutical Co., Ltd., 1-135, Komakado, Gotemba, Shizuoka 412-8513, Japan.

[2]ConfometRx, 3070 Kenneth St, Santa Clara, California 95054, USA

[3]Quantitative Biology and [4]Diabetes and Complications, Lilly Research Laboratories, Eli Lilly and Company, Indianapolis, Indiana 46285, USA

[¶]These authors contributed equally

*Corresponding authors: suzukiysy@chugai-pharm.co.jp; kobilka@stanford.edu; sloop_kyle_w@lilly.com

[#]Contributed by Brian K. Kobilka: kobilka@stanford.edu

Note: the present address for YS is: Translational Research Division, Chugai Pharmaceutical Co., Ltd., 2-1-1 Nihonbashi-Muromachi, Chuo-ku, Tokyo 103-8324, Japan.

www.pnas.org/cgi/doi/10.1073/pnas.2014879117

Supplemental Table and Figures

Supplemental Table 1

	$[^{125}I]$GLP-1(7-36) Binding	GLP-1R β-arrestin2		GLP-1R Low Density cAMP		GLP-1R Medium Density cAMP		GLP-1R High Density cAMP	
	Ki nM (SEM, n)	EC_{50} nM (SEM, n)	E_{MAX} % (SEM, n)	EC_{50} nM (SEM, n)	E_{MAX} % (SEM, n)	EC_{50} nM (SEM, n)	E_{MAX} % (SEM, n)	EC_{50} nM (SEM, n)	E_{MAX} % (SEM, n)
GLP-1(7-36)	0.779 (0.02, 116)	2.62 (0.25, 54)	104.2 (1.8, 54)	0.331 (0.007, 436)	105.5 (0.4, 436)	0.070 (0.020, 317)	104 (0.5, 317)	0.00625 (0.00013, 347)	107.9 (0.47, 347)
LY3502970	3.22 (1.4, 4)	>50,000 (NA, 3)	NA	3.05 (1.5, 3)	24.5 (1.6, 3)	1.38 (0.26, 14)	59.8 (2.9, 14)	0.494 (0.051, 12)	119.4 (3.3, 12)
TT-OAD2	19.0 (8.1, 3)	ND	ND	>50,000 (NA, 3)	NA	35.2 (13, 5)	10.8 (2.1, 5)	16.3 (2.6, 4)	52.8 (4.1, 4)

***In vitro* pharmacology of LY3502970 at the GLP-1R.** The affinity (K_i) of ligands for the GLP-1R was quantified by $[^{125}I]$GLP-1(7-36) competition binding using GLP-1R expressing cell membranes. Functional potency (EC_{50}) and efficacy (E_{MAX}) for β-arrestin recruitment using enzyme-fragment complementation was determined in CHO-K1 cells. GLP-1R density-dependent pharmacology of ligands was quantified by measuring the potency (EC_{50}) and efficacy (E_{MAX}) for cAMP accumulation in HEK293 cells at increasing levels of receptor density designated low (1,400 receptors/cell), medium (7,800 receptors/cell), and high density (76,000 receptors/cell). ND= Not Determined, NA= Not Applicable.

Supplementary Table 2. Cryo-EM data collection, refinement and validation statistics

	GLP-1R/LY3502970/G_{siN18}/Nb35/scFv16 (EMD-22283) (PDB-6XOX)
**Data collection and processing**	
Magnification	130,000
Voltage (kV)	300
Electron exposure (e–/Å^2)	80.9
Defocus range (μm)	-1.2 to -2.5
Pixel size (Å)	1.04
Symmetry imposed	C1
Initial particle images (no.)	1,598,390
Final particle images (no.)	583,053
Map resolution (Å)	3.2
FSC threshold	0.143
Map resolution range (Å)	2.8Å to 4.0Å
**Refinement**	
Initial model used (PDB code)	6VCB
Model resolution (Å)	3.5
FSC threshold	0.5
Map sharpening *B* factor (Å^2)	-109.5
B factors (Å^2)	91.7 (10,630 atoms)
GLP-1R	113.1 (380 residues, 2,989 atoms)
Ligand	103.9 (1 polymer, 65 atoms)

R.m.s. deviations	
Bond lengths (Å)	0.011
Bond angles (°)	1.08
Validation	
MolProbity score	1.69
Clashscore	6.38
Poor rotamers (%)	0.99
Ramachandran plot	
Favored (%)	95.1
Allowed (%)	4.9
Disallowed (%)	0

Supplemental Figure 1

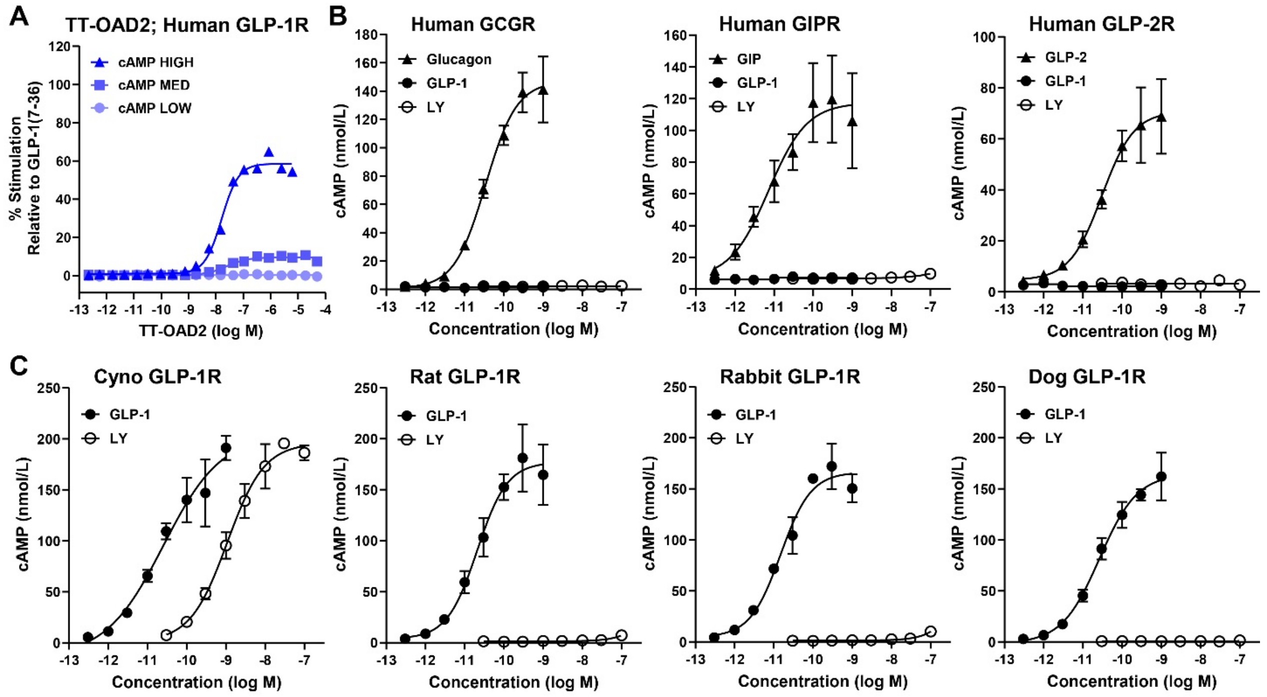

Supplemental Figure 1. Partial agonism of TT-OAD2 and LY3502970 effects on GLP-1R species specificity and class B GPCR selectivity. (A) Human GLP-1R density-dependent pharmacology of TT-OAD2 was quantified by measuring the potency and efficacy for cAMP accumulation at increasing levels of receptor density (high, medium, low). (B) For various class B GPCRs, cAMP accumulation by the cognate receptor ligand or LY3502970. (C) cAMP accumulation by GLP-1(7-36) or LY3502970 in HEK293 cells expressing high densities of either the cynomolgus monkey, rat, rabbit, or dog GLP-1R. Only the cynomolgus monkey GLP-1R contains Trp33^{ECD} as all others have Ser33^{ECD}. Panel A: representative concentration response curves of at least two independent experiments are presented. Panel B-C: Data are represented as the mean ± s.d. of three independent experiments.

Supplemental Figure 2

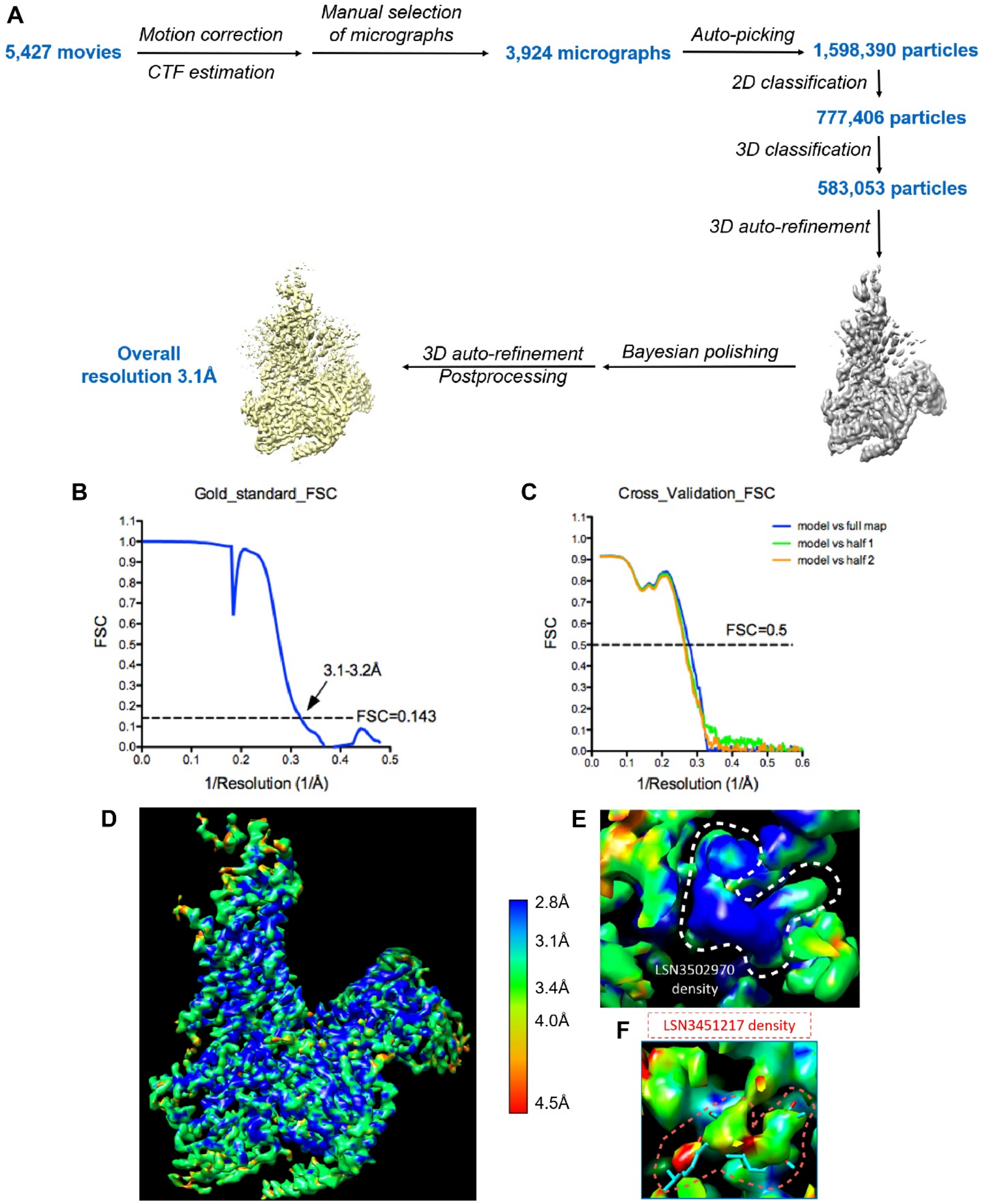

Supplemental Figure 2. Cryo-EM data processing and validation. (A) Cryo-EM data processing flow chart. (B) Gold standard Fourier shell correlation (FSC) curves of two individual half maps, indicating an average resolution of 3.1-3.2 Å at 0.143 FSC threshold. (C) Cross-validation of model to cryo-EM density map. The model was refined against one half map, and FSC curves were calculated between the outcome model and the half map used for refinement (green), the final cryo-EM map (full map, blue), and the other half map (orange). (D) Density map colored by local resolution. (E) The local resolution of the LY3502970 surrounding region. LY3502970 density is indicated by red dashed line. (F) The local resolution of LSN3451217 surrounding region. LSN3451217 density is indicated by orange dashed line. The density is too weak to allow confident modeling of the compound in the final model and a rough pose is shown here in stick.

Supplemental Figure 3

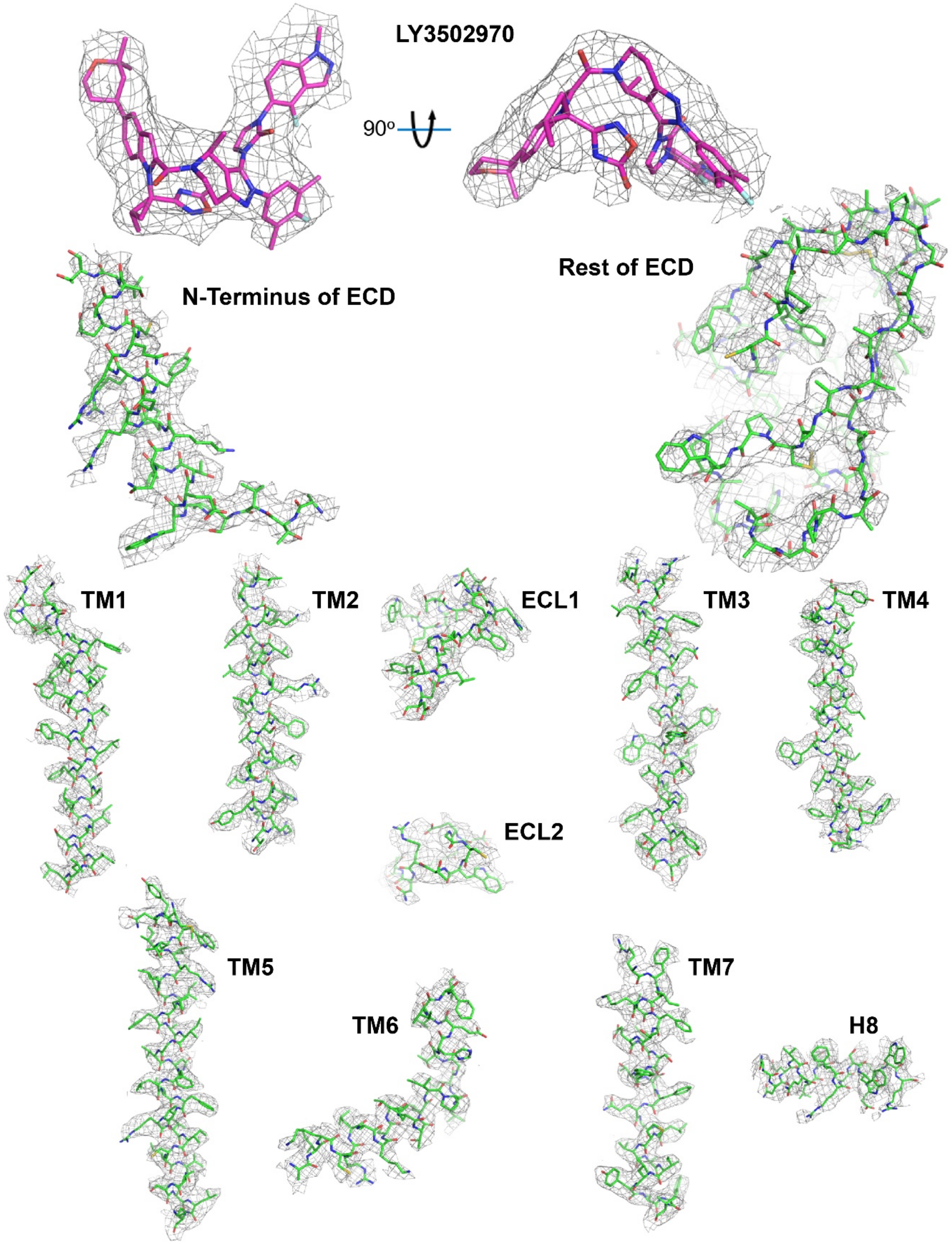

Supplemental Figure 3. Model of the GLP-1R/LY3502970/G_{siN18}/Nb35/scFv complex in the cryo-EM density map. The cryo-EM density map and model are shown for LY3502970 (zoomed in), extracellular domain (ECD), all seven transmembrane (TM) helices, extracellular loops 1 and 2 (ECL1, ECL2), and helix 8 (H8) of the GLP-1R.

Supplemental Figure 4

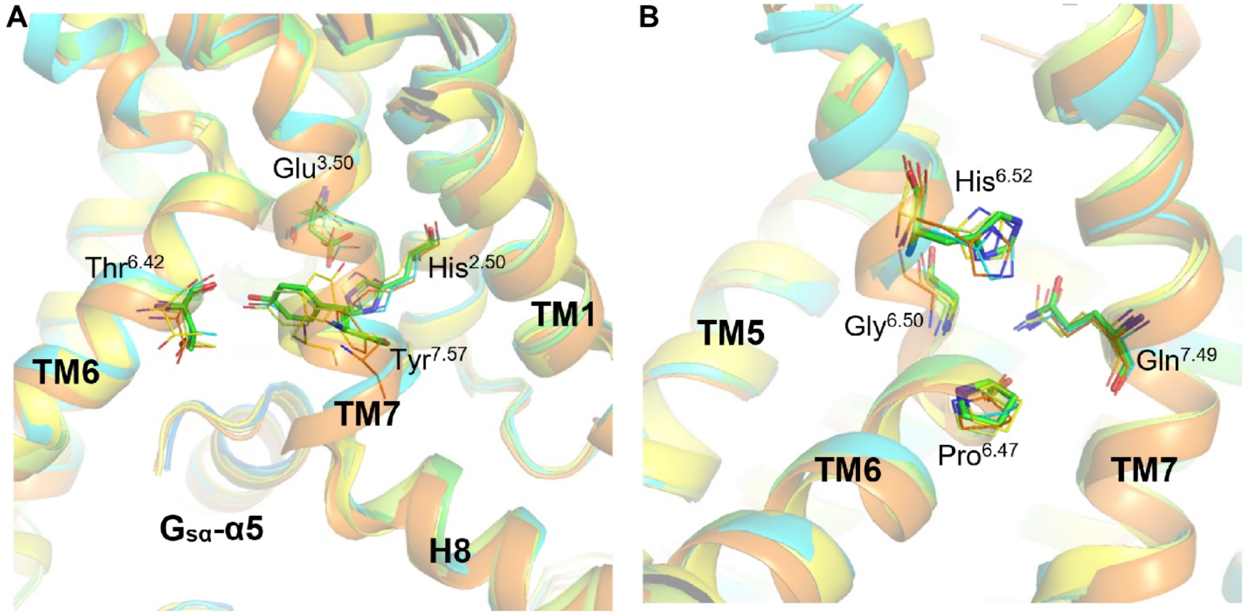

Supplemental Figure 4. Active state conformation of the GLP-1R. Multiple structures of active state GLP-1R are superimposed. (A) HETX motif and GLP-1R/Gs coupling interface. (B) PXXG motif and part of central polar network. The structures are colored as following: GLP-1R bound to LY3502970 (green for GLP-1R and blue for Gs); bound to GLP-1 (PDB ID: 5VAI; lemon green); bound to GLP-1 and a positive allosteric modulator LSN3160440 (PDB ID: 6VCB; lemon green); bound to ExP5 (PDB ID: 6B3J; orange); bound to TT-OAD2 (PDB ID: 6ORV; cyan). Residues from LY3502970 bound GLP-1R structure are shown in sticks and residues from other structures are shown in lines.

Supplemental Figure 5

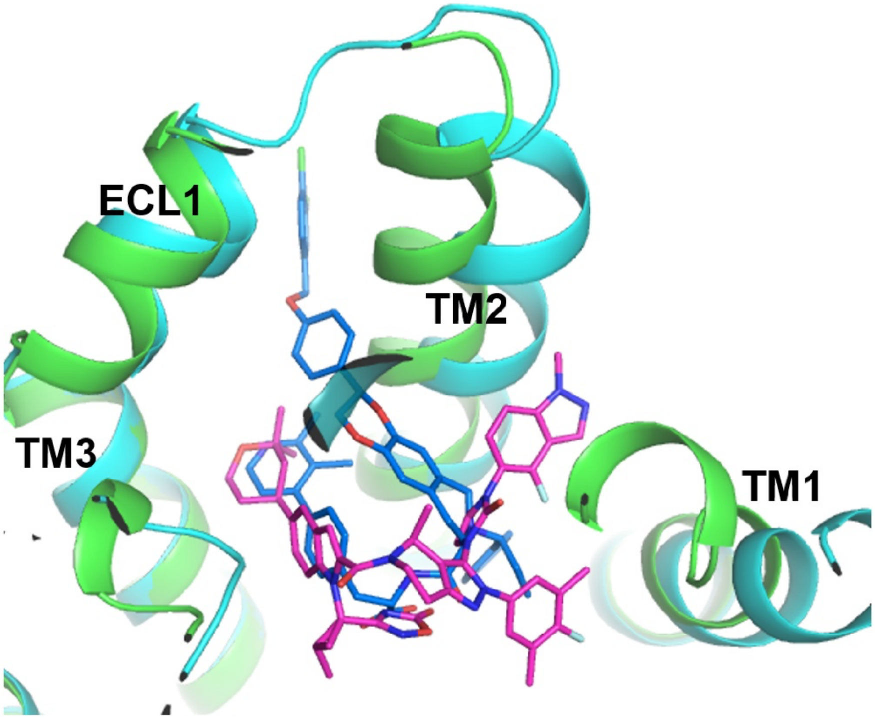

Supplementary Fig. 5. Binding poses of LY3502970 and TT-OAD2 to GLP-1R. The structures of GLP-1R bound to LY3502970 (receptor in green, LY3502970 in magenta) and bound to TT-OAD2 (receptor in cyan, TT-OAD2 in blue, PDB ID: 6ORV) are aligned. The receptors are shown in cartoon and ligands are shown in sticks.

Supplemental Figure 6

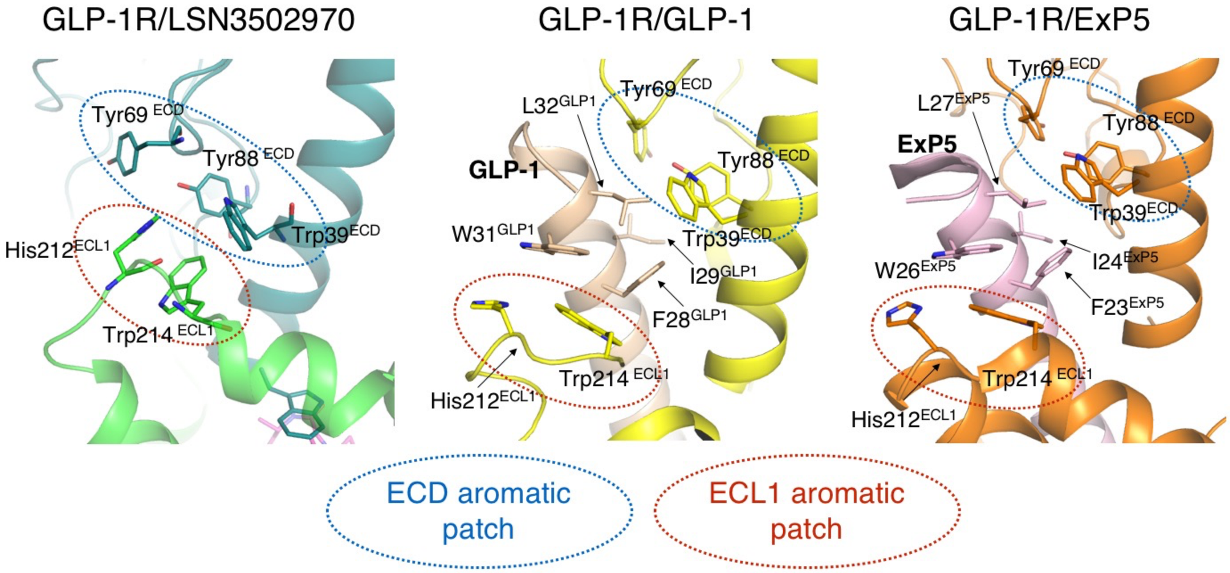

Supplemental Figure 6. The unique GLP-1R ECD orientation in the LY3502970 bound structure is stabilized by aromatic interactions with ECL1. The structures of GLP-1R bound to LY3502970 (7TM in green, ECD in blue green), bound to GLP-1 (receptor in yellow, GLP-1 in beige, PDB ID: 6VCB) and bound to ExP5 (receptor in orange, ExP5 in pink, PDB ID: 6B3J) are shown. Important aromatic and hydrophobic residues are shown in sticks.

Supplemental Figure 7

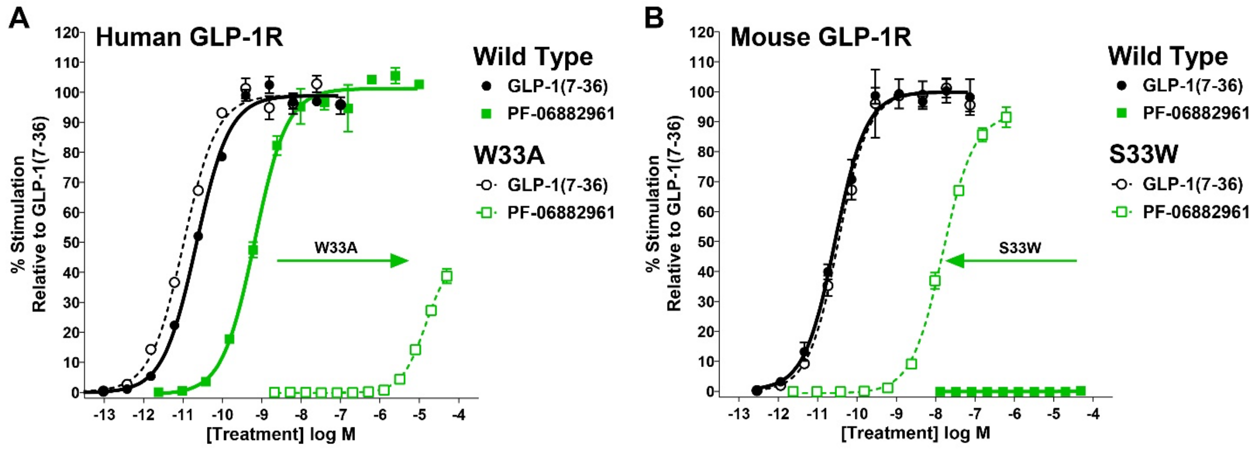

Supplemental Figure 7. Activation of the GLP-1R by the non-peptide agonist PF-06882961 requires Trp33^{ECD}. (A) Using high GLP-1R density HEK293 cells, mutation of Trp33^{ECD} to Ala33^{ECD} in the human GLP-1R abolishes activity of PF-06882961. (B) Mutation of Ser33^{ECD} to Trp33^{ECD} in the mouse GLP-1R enables PF-06882961 to induce receptor signaling. For each panel, representative concentration response curves of at least two independent experiments are presented.

Supplemental Figure 8

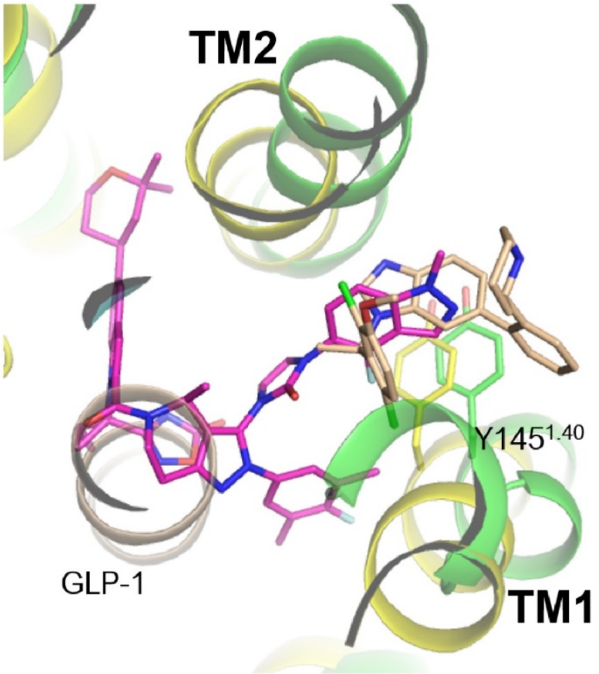

Supplemental Figure 8. The 4-fluoro-1-methyl-indazole moiety of LY3502970 occupies a similar position to that of the positive allosteric modulator LSN3160440. The two structures of GLP-1R bound to LY3502970 (green for GLP-1R and magenta for LY3502970) or bound to GLP-1 and the positive allosteric modulator LSN3160440 (PDB ID: 6VCB; lemon green) are superimposed. The small molecule ligands and Tyr145$^{1.40}$ in both structures are shown in sticks.

Supplemental Figure 9

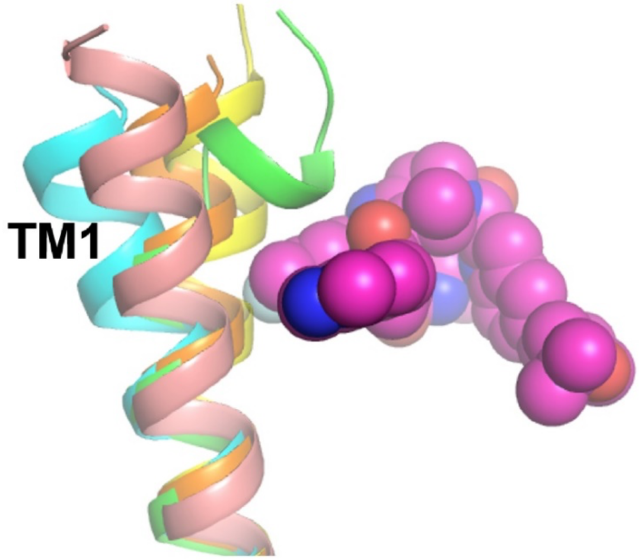

Supplemental Figure 9. The unique TM1 kink induced by LY3502970 binding. The structures of TM1 of GLP-1R bound to LY3502970 (7TM in green; LY3502970 in magenta sphere), TT-OAD2 (cyan, PDB ID: 6ORV), GLP-1 (yellow, PDB ID: 6VCB), ExP5 (orange, PDB ID: 6B3J) and inactive state structure of GLP-1R (salmon, PDB ID: 6LN2) are aligned. Only TM1 of GLP-1R bound to LY3502970 has a kink at the amino terminus. Other peptides bound GLP-1R structures (GLP-1, PDB ID: 5VAI; peptide 5, PDB ID: 5NX2) are not shown here but their TM1 conformations are very similar to GLP-1 bound structure 6VCB in yellow.

Supplemental Figure 10

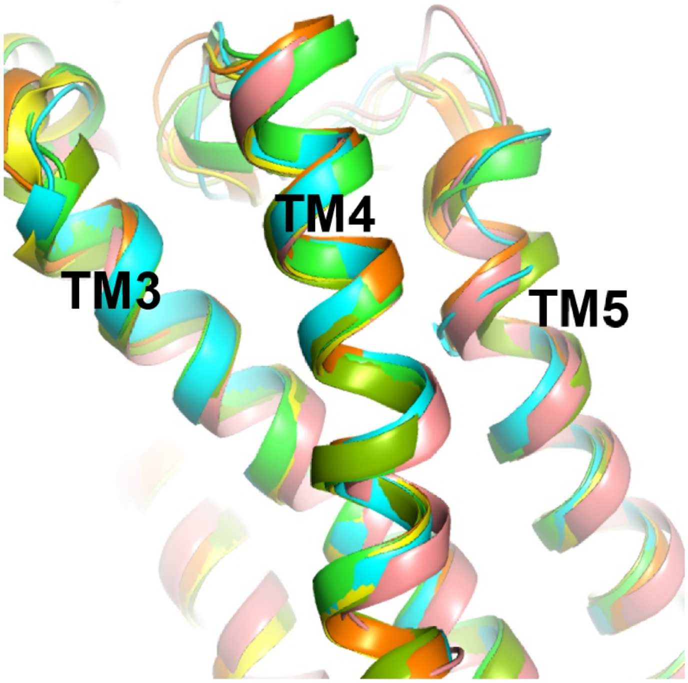

Supplemental Figure 10. The conformation of TM3, TM4, and TM5 are similar among various GLP-1R structures. The structures of GLP-1R bound to LY3502970 (7TM in green, LY3502970 in magenta sphere), GLP-1 (yellow, PDB ID: 6VCB; lemon green, PDB ID: 5VAI), ExP5 (orange, PDB ID: 6B3J), peptide 5 (gray, PDB ID: 5NX2), TT-OAD2 (cyan, PDB ID: 6ORV) and antagonist PF-06372222 (salmon, PDB ID: 6LN2) are aligned.
